# Carbon ions induce autophagy effectively through stimulating the unfolded protein response and subsequent inhibiting Akt phosphorylation in tumor cells

**DOI:** 10.1038/srep13815

**Published:** 2015-09-04

**Authors:** Xiaodong Jin, Feifei Li, Xiaogang Zheng, Yan Liu, Ryoichi Hirayama, Xiongxiong Liu, Ping Li, Ting Zhao, Zhongying Dai, Qiang Li

**Affiliations:** 1 Institute of Modern Physics, Chinese Academy of Sciences, Lanzhou 730000, China; 2 Key Laboratory of Heavy Ion Radiation Biology and Medicine of Chinese Academy of Sciences, Lanzhou 730000, China; 3 University of Chinese Academy of Sciences, Beijing 100049, China; 4 Research Center for Charged Particle Therapy, National Institute of Radiological Sciences, Anagawa 4-9-1, Inage-ku, Chiba 263-8555, Japan

## Abstract

Heavy ion beams have advantages over conventional radiation in radiotherapy due to their superb biological effectiveness and dose conformity. However, little information is currently available concerning the cellular and molecular basis for heavy ion radiation-induced autophagy. In this study, human glioblastoma SHG44 and cervical cancer HeLa cells were irradiated with carbon ions of different linear energy transfers (LETs) and X-rays. Our results revealed increased LC3-II and decreased p62 levels in SHG44 and HeLa cells post-irradiation, indicating marked induction of autophagy. The autophagic level of tumor cells after irradiation increased in a LET-dependent manner and was inversely correlated with the sensitivity to radiations of various qualities. Furthermore, we demonstrated that high-LET carbon ions stimulated the unfolded protein response (UPR) and mediated autophagy via the UPR-eIF2α-CHOP-Akt signaling axis. High-LET carbon ions more severely inhibited Akt-mTOR through UPR to effectively induce autophagy. Thus, the present data could serve as an important radiobiological basis to further understand the molecular mechanisms by which high-LET radiation induces cell death.

Heavy ion therapy, also called carbon ion therapy, is becoming an increasingly important option for radiation cancer therapy^1^,^2^. Up to now, more than 14,000 patients have been treated with carbon ions, demonstrating the advantage of carbon ion radiotherapy over other modalities for various types of tumors in terms of high local control and survival rates^3^ (http://www.ptcog.ch). Charged particles, such as carbon, neon and other heavy ions, demonstrate an increase in energy deposition with penetration depth up to a sharp maximum at the end of their range, known as the Bragg peak^4^. This feature makes heavy ion beams possess an excellent dose distribution, allowing precise localization of a sufficient dose in the target lesion while minimizing the damage to the surrounding normal tissues. Another advantage of charged particles over X-rays is their higher linear energy transfer (LET). Compared with sparsely ionizing radiation such as X-rays, high-LET particle radiation has a higher relative biological effectiveness (RBE), reduced oxygen enhancement ratio (OER) and nearly unchanged radiosensitivity within the cell cycle^1^,^5^. High-LET radiation can also selectively target cancer stem cells *in vitro* and *in vivo*^6^.

Many efforts have been made to decipher the molecular mechanisms by which high-LET radiation induces cell death in cancer cells. Recently, some reports have revealed a critical cell response to severe environments, such as starvation, hypoxia, and reactive oxygen species (ROS), which is called macroautophagy (referred to hereafter as autophagy). Autophagy is an evolutionarily conserved process by which cells recycle their components, such as long-lived proteins and damaged organelles, and involves the sequestration of cytoplasmic components within a double-membrane structure termed the autophagosome and their subsequent delivery to lysosomes for degradation^7^,^8^,^9^. We and others have already reported high-LET carbon ion-induced autophagy in tumor cells^10^,^11^,^12^. We found that carbon ions could effectively elicit autophagy flux in tumor cells, and the autophagic level increased in a LET-dependent manner^12^. However, the corresponding mechanisms underlying these observations are still poorly understood.

In this study, we investigated the dependence of autophagy on the LET of radiation. Furthermore, the molecular mechanisms of the autophagy response to different LET radiations in diverse signaling pathways were explored. For the first time, in the present study, we revealed why autophagic levels increase in a LET-dependent manner in irradiated tumor cells.

## Results

### Carbon ions induce autophagy in a LET- and dose-dependent manner in tumor cells

**Figure 1:**
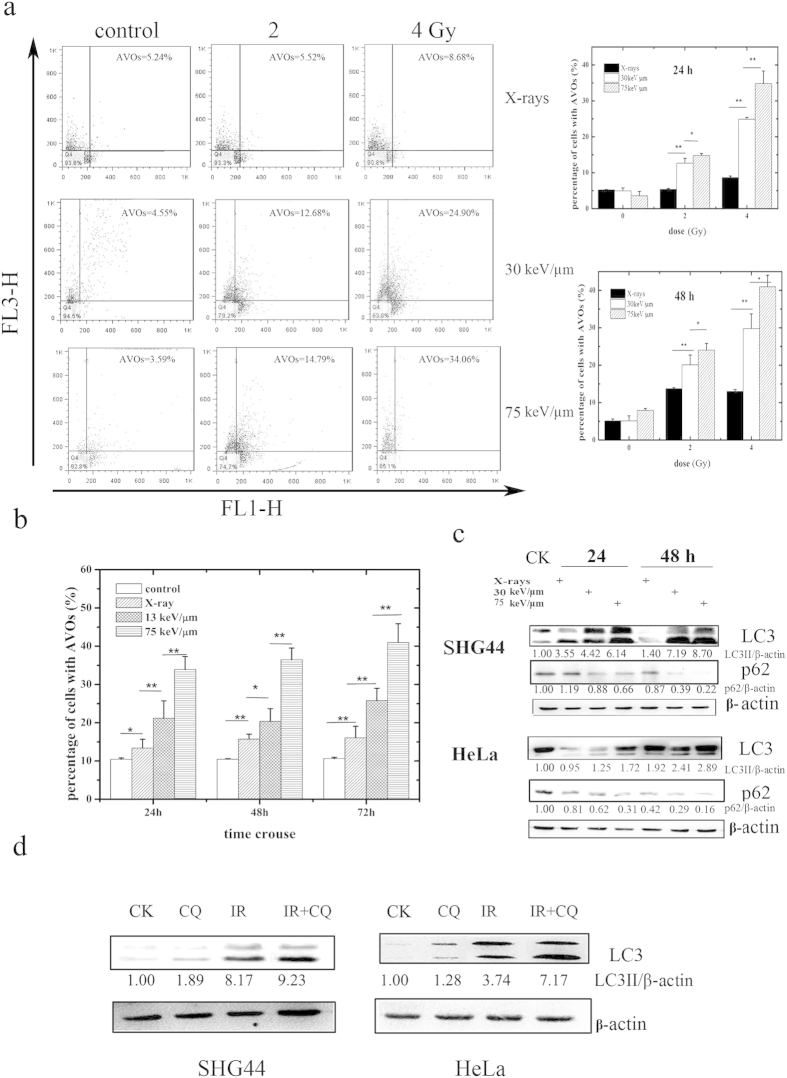
Autophagy induced by carbon ions with different LETs. (**a**) Quantification of autophagy by flow cytometry in SHG44 cells. Representative images of flow cytometry at 24 h after irradiation are presented on the left, where FL1-H (normal cells) and FL3-H (cells with autophagosomes) indicate green and red color intensities, respectively. The right figures show the statistical results of three independent experiments at 24 and 48 h post-irradiation. (**b**) The statistical results of autophagy levels using flow cytometry in HeLa cells (dose = 5 Gy). The data for carbon ions were published in our previous paper^12^. (**c**) LC3-II conversion and the expression of p62 in SHG44 and HeLa cells after exposure to a radiation dose of 2 Gy. (**d**) Pretreatment with CQ inhibited the autophagic flux in irradiated cells with carbon ions of 75 keV/μm at 2 Gy. (*p < 0.05, **p < 0.01 in (**a**,**b**)).

Autophagy induction in SHG44 and HeLa cells exposed to X-rays and carbon ions of different LETs, as indicated by the presence of acidic vesicular organelles (AVOs), was explored in this study. Figure 1a shows the autophagy levels in SHG44 cells irradiated with X-rays and carbon ions with LETs of 30 and 75 keV/μm at a dose of 2 Gy or 4 Gy at 24 h and 48 h post-irradiation. Clearly, the autophagic rate of the SHG44 cells increased in a LET- and dose-dependent manner at the indicated time points after carbon ion irradiation. Similar results were observed in HeLa cells (Fig. 1b).

The conjugation of the soluble form of LC3 (LC3-I) with phosphatidylethanolamine (PE) and its conversion into a non-soluble form (LC3-II) is a hallmark of autophagy^13^; thus, we examined the expression of LC3-II. After irradiation at the indicated time points, LC3-II levels increased in the two cell lines in a LET-dependent manner (Fig. 1c). Moreover, the irradiation down-regulated the expression of the natural autophagic substrate p62^13^, and this decrease also occurred in a LET-dependent manner in these two cell lines. To monitor the autophagic flux induced by the carbon ions, we co-treated both cell lines with radiation and chloroquine (CQ) (10 μM), which blocks the downstream steps of autophagy. The co-treatment increased the conversion of LC3-II compared with radiation alone (Fig. 1d), suggesting that the carbon ions elicited complete autophagy. Collectively, these data confirmed that irradiation-induced autophagy in SHG44 and HeLa cells and the autophagic levels of irradiated cells increased in a LET- and dose-dependent manner.

### The relationship between autophagy and cell survival after exposure to X-rays and carbon ions of different LETs

**Figure 2:**
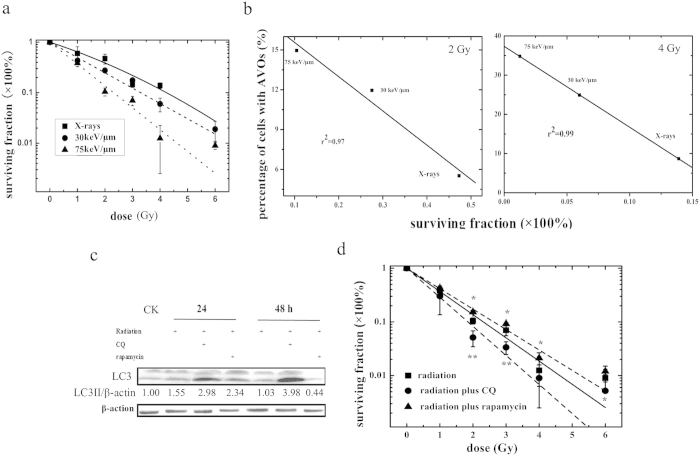
The relationship between autophagy and cell survival after exposure to X-rays and carbon ions of different LETs. (**a**) Survival curves of SHG44 cells exposed to X-rays and carbon ions. (**b**) The AVO levels in SHG44 cells 24 h after irradiation as a function of survival fraction. The data were fitted using the least-squares method. (**c**) The co-treatment of 75 keV/μm carbon ions at 2 Gy with CQ or rapamycin inhibited or enhanced LC3-II expression. (**d**) Effect of pharmacological inhibition or promotion of autophagy on the sensitivity of SHG44 cells to high-LET radiation (75 keV/μm carbon ions) (*p < 0.05, **p < 0.01, co-treatment with radiation and drug versus radiation alone).

To examine the effect of carbon ions of different LETs and X-rays on the survival of tumor cells, SHG44 cells were irradiated at doses varying from 1 to 6 Gy. Figure 2a shows the corresponding survival curves of SHG44 cells after exposure to X-rays and carbon ions. The parameters of the survival curves and α and β coefficients that were used in the linear quadratic (LQ) model^5^ are summarized in Table S1. The survival curve for the X-rays was fitted well by the LQ model, having both α and β terms. However, for the carbon ions, the survival curves were fitted well by the LQ equation without the β term (or β = 0) because the curves were linear in the semi-logarithmic coordinate. Compared with X-rays, cells that were exposed to carbon ions showed higher radiosensitivities without shoulders, and their sensitivities increased along with the LET values of their carbon ions. The doses at D_10_ (the dose required to reduce the cell survival fraction to 10%) were approximately 2.3 Gy (75 keV/μm, carbon ions), 3.3 Gy (30 keV/μm, carbon ions) and 4.2 Gy (X-rays). The RBE values of the D_10_ values of the carbon ions of 30 and 75 keV/μm were 1.3 and 1.8, respectively.

To clarify the relationship between radiosensitivity and autophagy, the cell-surviving fractions were plotted against the level of autophagy at 24 h after irradiation, as shown in Fig. 2b. The surviving fraction data were correlated well with the level of autophagy at each radiation dose (correlation coefficient r^2^ = 0.97 for 2 Gy and 0.99 for 4 Gy). A close correlation between survival and autophagy levels in response to radiation was observed, indicating that autophagy plays a role in cellular radiosensitivity.

Although our results suggested that autophagy level is related to radiosensitivity to carbon ion radiation in SHG44 cells, the question of whether autophagy induced by high-LET radiation represents a survival mechanism or contributes to cell death remains. To explore the role of autophagy in high-LET radiation-induced cytotoxicity in tumor cells, CQ, which prevents lysosomal acidification to block autophagic catabolism, and rapamycin, a potent inhibitor of mTOR that has been shown to enhance autophagy^14^, were used in our study. As shown in Fig. 2c, there were increased levels of LC3-II expression in CQ- or rapamycin-pretreated SHG44 cells irradiated with carbon ions. The effects of these reagents on the clonogenic survival of SHG44 cells irradiated with carbon ions is exhibited in Fig. 2d. The surviving fraction of SHG44 cells was reduced significantly in the presence of CQ. By contrast, rapamycin pretreatment led to higher autophagic rates and increased survival fractions compared with treatment with irradiation alone. Interestingly, we also found that the percentage of cells with AVOs that were closely correlated with the survival fraction of treated cells differed at 2 Gy (correlation coefficient r^2^ = 0.95), as shown in Figure S1. All of the data shown above indicated that autophagy might play an important role in radiosensitivity, promoting cell survival and contributing to cellular resistance against high-LET radiation.

### Carbon ions induces autophagy in tumor cells by inhibiting the Akt-mTOR pathway

**Figure 3:**
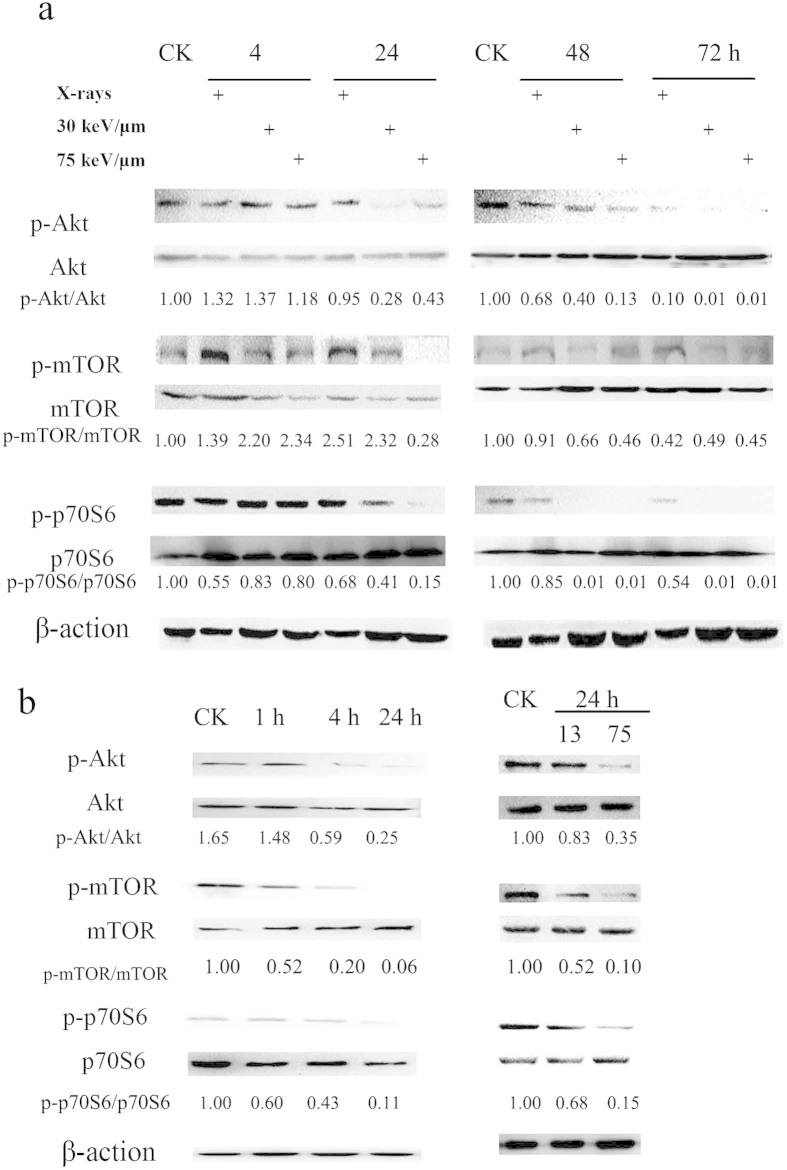
The Akt-mTOR signaling pathway was effectively decreased by X-rays or carbon ions with different LETs. (**a**) SHG44 cells were irradiated at 2 Gy. (**b**) HeLa cells were exposed to carbon ions of 75 keV/μm at 2 Gy (left) or different LETs at 2 Gy (right). The data concerning the phosphorylation of proteins in HeLa cells were published in our previous paper^12^.

To explore the molecular mechanisms underlying the changes in the levels of autophagy that are induced by X-rays or carbon ions with different LETs in tumor cells, the Akt-mTOR pathway was evaluated in SHG44 and HeLa cells. mTOR acts as a central regulator in autophagy induction, and Akt modulates the activation of mTOR^15^. Thus, we investigated whether radiation-induced autophagy occurred via mTOR inhibition. The levels of phospho-Akt (p-Akt), phosphor-mTOR (p-mTOR) and phospho-p70S6 (p-p70S6), all of which are mTOR substrates, in the two tumor cell lines were measured. In SHG44 cells, the activation of p-Akt was slightly increased at 4 h after irradiation with radiations of various qualities. At 24 h post-irradiation, the high-LET carbon ions caused an obvious decrease in the level of p-Akt proteins compared with the X-rays. The activation of p-Akt in cells exposed to X-rays was also decreased at 48 and 72 h post-irradiation, but to a much lower extent in carbon ion-irradiated cells. Similar to p-Akt, the activation levels of p-mTOR and p-p70S6 were decreased in SHG44 cells in a time- and LET-dependent manner. As shown in Fig. 3b, high-LET radiation decreased the activation of the Akt-mTOR pathway in HeLa cells as well. Moreover, greater reductions in the expression levels of p-Akt, p-mTOR and p-p70S6 were observed in HeLa cells after their exposure to carbon ions of 75 keV/μm than after exposure to carbon ions of 13 keV/μm. These results indicated that carbon ion radiation might elicit autophagy in tumor cells via decreasing the activation of the Akt-mTOR pathway, and this pathway was more effectively inhibited by carbon ions with high LETs than relatively low LETs.

### Carbon ions induce autophagy via activation of endoplasmic reticulum (ER) stress

**Figure 4:**
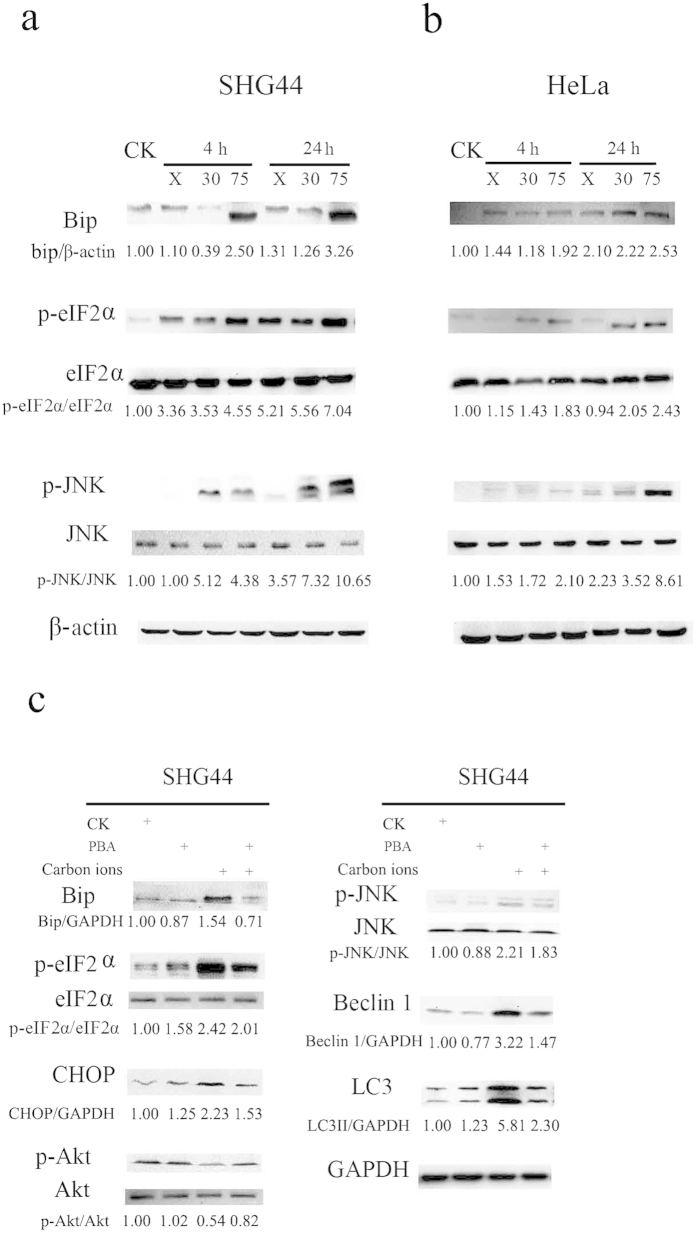
Radiation-induced autophagy via UPR. Bip and the key molecules of autophagy were upregulated by carbon ions of different LETs at 2 Gy in SHG44 (**a**) and HeLa cells (**b**). (**c**) PBA treatment prevented the activation of UPR and inhibited autophagy induced by carbon ions in SHG44 cells.

Because ER stress is one of the signaling pathways involved in the regulation of autophagy^16^, we hypothesized that ER stress might also play an important role in radiation-induced autophagy. SHG44 and HeLa cells were irradiated with X-rays and carbon ions of 30 and 75 keV/μm at 2 Gy. The expression levels of Bip, a major indicator of UPR^17^, at 4 and 24 h post-irradiation are shown in Fig. 4a,b for SHG44 and HeLa cells, respectively. Clearly, the expression levels of Bip were promoted by radiation, indicating the occurrence of ER stress in tumor cells after irradiation. It is well known that eIF2α and JNK are involved in autophagy induction through UPR^18^,^19^. The phosphorylated levels of these two types of proteins increased in both a LET-dependent and time-dependent manner (Fig. 4a,b), suggesting that carbon ion radiation-activated UPR might be involved in autophagy induction in tumor cells. Next, we examined whether the inhibition of UPR using 4-phenylbutyric acid (PBA) influenced the autophagy level in SHG44 cells exposed to the carbon ions of 75 keV/μm. PBA acts as a chemical chaperone in the ER to prevent the activation of UPR and inhibit ER stress^20^. In mock cells, PBA did not alter the levels of Bip, p-Akt, phospho-JNK (p-JNK) or Beclin 1, but it slightly enhanced the expression levels of phospho-eIF2α (p-eIF2α), CHOP and LC3-II (Fig. 4c, lines 1 and 2). As shown in Fig. 4c, the carbon ions upregulated the expression of these proteins (line 3). However, PBA rescued the carbon ion-induced UPR (line 4), as indicated by decreased Bip and CHOP expression and phosphorylation of JNK compared to irradiation with carbon ions alone. Interestingly, the Akt phosphorylation level recovered completely. PBA also decreased the expression levels of Beclin 1, p-eIF2α and LC3-II, but the levels were not reversed to basal conditions. Therefore, these results indicated that high-LET carbon ions effectively inhibited Akt phosphorylation and increased Beclin 1 expression under UPR, resulting in autophagy enhancement.

## Discussion

Recently, heavy ion radiotherapy has emerged as a promising new anticancer strategy, and many studies have been undertaken to explore the molecular mechanisms underlying its anticancer action. Although our previous work supported that autophagic level increased in a LET-dependent manner in tumor cells^12^, in this study, for the first time, we confirmed that exposure to radiations of various qualities influences the levels of autophagy in tumor cells at the molecular level. As one of three principal methods of assessment^21^, the membrane-associated form of LC3, which is converted from LC3-I to LC3-II, is widely used to monitor the number of autophagosomes. In addition to LC3, p62 can also be used as a protein marker, and decreased p62 levels are associated with autophagy activation^13^. In our study, low-LET X-rays elicited a slight increase in the expression level of LC3-II protein, whereas LC3-II expression after heavy ion exposure depended on the LET values of carbon ions in SHG44 and HeLa cells (Fig. 1c). In contrast, p62 expression was suppressed in a LET-dependent manner in these two cell lines (Fig. 1c). These results were also verified by flow cytometry measurements (Fig. 1a,b).

Consistent with previous studies^5^,^22^, we observed that high-LET carbon ions had a more pronounced effect on survival ability than low-LET X-rays, and the radiosensitivity of SHG44 cells to carbon ions changed in a LET-dependent manner. We further analyzed the relationship between clonogenic survival and cell autophagic level after irradiation with different LETs at 2 and 4 Gy in SHG44 cells. The increase in the surviving cell fraction was inversely proportional to the autophagic level. To our knowledge, this is the first study to reveal a correlation between cellular radiosensitivity and autophagy, which might be used to predict radiosensitivity in biopsy cultures exposed to radiations of different qualities. To gain a deeper understanding of the role of autophagy in cytotoxicity induced by high-LET radiation, we next investigated whether manipulating autophagy could influence the radiosensitivity of tumor cells. We found that both cell death and radiosensitivity were remarkably enhanced when combined with autophagy inhibitor treatment. Conversely, cells that were co-treated with irradiation and rapamycin had an increased resistance to carbon ions, suggesting that autophagy contributed to promoting the survival of tumor cells irradiated with high-LET carbon ions. These results, which were obtained from autophagy-manipulation experiments, coincided with previous studies in which autophagy inhibition was shown to increase cellular sensitivity to various therapies^23^,^24^,^25^,^26^.

Additionally, a question arose concerning how high-LET radiation induced a more severe autophagy effect than low-LET X-rays at the same dose. Next, two classic molecular pathways, the Akt-mTOR and UPR pathways, were investigated. mTOR is a potent repressor of autophagy, interacting with the ULK1 kinase complex and directly phosphorylating the ATG13L and ULK1 subunits to repress ULK1 kinase activity^27^,^28^. Li *et al.* indicated that plumbagin, a natural naphthoquinone, causes autophagy via inhibition of the Akt-mTOR pathway in A549 cells^29^. Luteolin induced autophagy in squamous cell carcinoma cells and also inhibited the activation of the Akt-mTOR-p70S6K pathway^30^. Here, we observed that carbon ions apparently inhibited the Akt-mTOR pathway efficiently and reduced the expression of p-mTOR, depending on their LETs. These results are consistent with a report by Nakagawa *et al.*^31^ that showed that carbon ion beam irradiation effectively suppresses mTOR and associated proteins compared with low-LET radiation.

In addition to the Akt-mTOR pathway, ER stress may also induce autophagy. Various physiological and pathological conditions may cause the accumulation of unfolded or misfolded proteins, resulting in a cellular adaptive procedure known as ER stress^32^, which triggers the UPR as an adaptive response to ensure cell survival or to mediate cell death if the stress is too severe^16^,^17^. Zhang *et al.* found that X-rays induced the upregulation of ER stress markers, including Bip and GRP94, at the protein and mRNA levels in IEC-6 cells^32^. Chiu *et al.* observed that increases in IRE1 and the phosphorylation of eIF2α after exposure to X-rays are correlated with DNA damage^33^. Consistent with this observation, we found that the expression of Bip, which is a sensor of ER stress^16^,^34^, was enhanced after irradiation, suggesting that both X-rays and high-LET radiation could elicit this effect in tumor cells. Furthermore, Bip expression increased in a time- and LET-dependent manner in response to carbon ions, indicating that high-LET radiation caused more severe ER stress than X-rays. Upon ER stress, Bip dissociates from the luminal domains of PERK and subsequently activates PERK. Activated PERK phosphorylates eIF2α and mediates autophagy via the ATF4-DDIT3/CHOP-TRIB3-Akt-mTOR axis^26^,^35^. In addition, Bip dissociation activates IRE1. Activated IRE1 can also recruit TNFR-associated factor 2 (TRAF2) to form the IRE1-TRAF2-ASK1 complex, which phosphorylates JNK. Subsequently, activated JNK phosphorylates Bcl-2 located in the ER, while Bcl-2 dissociates from Beclin 1 to trigger autophagy^36^. We examined the expression levels of the key proteins of these signaling pathways. As shown in Fig. 4a,b, along with an increase in LET, the phosphorylation of eIF2α and JNK in carbon ion-irradiated cells increased at the same dose (2 Gy). Moreover, PBA treatment rescued the UPR induced by carbon ions and led to the suppression of autophagy, implying that PERK and IRE1-mediated ER stress were required for autophagy induction by high-LET radiation.

Interestingly, we found that the phosphorylation level of Akt recovered to the basal level following the combined treatment of PBA and irradiation, while irradiation alone decreased the phosphorylation of Akt (Fig. 4c), indicating that PBA, as a specific UPR inhibitor, rescued Akt-mTOR activity. The recovery of Akt-mTOR activity might inhibit autophagy, as verified by LC3-II expression. This result is consistent with several reports in which ER stress inhibits the activation of the Akt pathway. Huang *et al.* reported that ER stress negatively regulates the Akt-TSC-mTOR pathway to enhance autophagy^35^. It has also been reported that the ER stress-ATF4-CHOP-TRIB3-Akt-mTOR pathway participates in salinomycin-induced LC3 conversion^26^.

**Figure 5:**
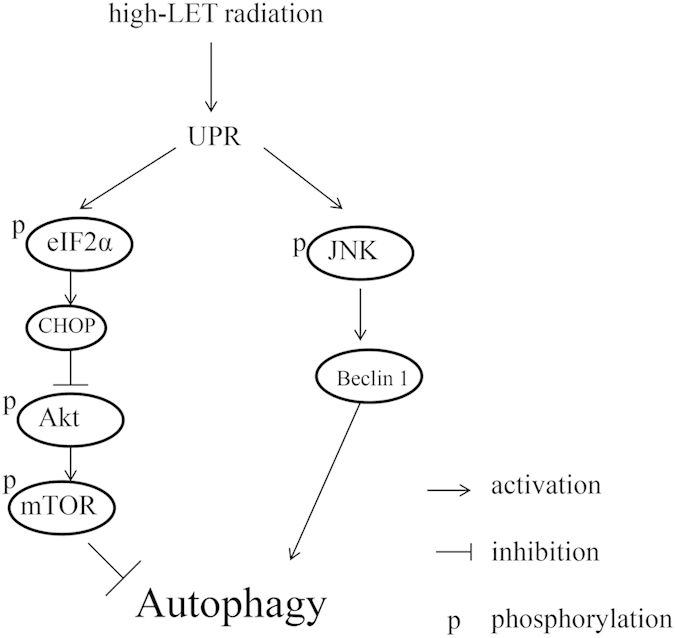
A proposed model of a molecular interaction to delineate the action mechanism of autophagy induced by high-LET radiation.

In addition, according to the Western blot results shown in Figs 3 and 4, the up-regulation of UPR started at 4 h post-irradiation; however, the levels of p-Akt, p-mTOR and p-p70S6 did not change at all at this time point; even a slight increase in p-Akt and p-mTOR levels. A down-regulation of Akt-mTOR was observed at 24 h after irradiation. Thus the up- and down-regulation occurred in a sequential order. Therefore we propose that Akt-mTOR is located downstream of the UPR pathway and that high-LET carbon ions activate UPR. The latter induces the phosphorylation of eIF2α and CHOP expression, which consequently inhibits Akt and mTOR activation via the blockade of Akt phosphorylation, leading to the occurrence of autophagy. Moreover, this process occurred in a LET-dependent manner. Notably, high-LET radiation also induced autophagy through the UPR-JNK-Beclin 1 pathway, as shown in Fig. 4. Based on the analyses above, the molecular mechanism of autophagy induced by high-LET radiation is summarized in Fig. 5.

In conclusion, the present study demonstrates that high-LET radiation can induce autophagy effectively in tumor cells, and the autophagic level increased in a LET-dependent manner. A positive correlation between cell survival and autophagy level was observed in tumor cells in response to radiations of various qualities. For the first time, we found that the high autophagic levels of tumor cells after exposure to high-LET radiation was due to more effective activation of the UPR pathway and subsequent p-Akt blockade compared with low-LET radiation. Thus, our data shed light on the mechanisms by which heavy ions elicit autophagy.

## Materials and Methods

### Reagents

CQ, rapamycin and PBA, purchased from Sigma-Aldrich Co. (St Louis, USA), were used at concentrations of 10 μM, 1 μM and 5 mM, respectively, in all experiments. Rapamycin and PBA were dissolved in dimethyl sulfoxide (DMSO), while CQ was dissolved in phosphate-buffered saline (PBS). These reagents at the corresponding concentrations were examined to be non-cytotoxic or less cytotoxic to the tumor cells used in this study (data not shown). The cells were pretreated with CQ and rapamycin for 4 h before irradiation and with PBA for 12 h before sample collection.

### Cell culture

The human glioblastoma cell line SHG44 was preserved in our laboratory. The human cervical cancer cell line HeLa was purchased from the Type Culture Collection of the Chinese Academy of Sciences (Shanghai, China). These two cell lines were maintained in RPMI-1640 medium supplemented with 100 U/ml penicillin, 100 μg/ml streptomycin and 10% (v/v) fetal bovine serum (Gibco, USA) and were kept at 37 °C and 5% CO_2_ in incubators.

### Irradiation

X-rays: Cells were plated in T25 flasks 24 h before irradiation and subsequently irradiated with X-rays, which were generated using an X-ray machine (FAXITRON RX-650, Faxitron Bioptics, LLC, Tucson, AZ, USA) operated at 50 kVp. The dose rate was approximately 0.5 Gy/min. All of the irradiations were carried out at room temperature.

Carbon ions: Most of the irradiations were performed with a carbon ion beam of 165 MeV/u in the heavy ion therapy terminal of the Heavy Ion Research Facility in Lanzhou (HIRFL) at the Institute of Modern Physics (IMP), Chinese Academy of Sciences. Some experiments were conducted with a carbon ion beam of 290 MeV/u in the Heavy Ion Medical Accelerator in Chiba (HIMAC) at the National Institute of Radiological Sciences (NIRS), Japan. Asynchronously growing cells were seeded in T25 flasks 24 h prior to exposure to carbon ions. The dose-averaged LET of the carbon ion beams on the cell samples was adjusted to 13, 30 or 75 keV/μm according to our experimental requirements. All of the irradiations were carried out at room temperature, and the control groups were sham-irradiated.

### Flow cytometry

Autophagy is characterized by the formation of acidic vesicular organelles (AVOs)^37^. To detect and quantify AVOs, cells were stained with 1.0 μg/ml acridine orange (AO) for 15 min. In the AO-stained cells, acidic compartments such as autophagosomes appeared bright red. Green (510–530 nm) and red (>650 nm) fluorescence, illuminated with blue (488 nm) light excitation, was measured using a flow cytometer (FACSCalibur, Becton-Dickinson, USA) and analyzed using FlowJo software (Treestar Inc., Ashland, OR, USA).

### Western blotting

Total cellular extracts were prepared as described previously^12^ and transferred to PVDF membranes. Blots were incubated with the indicated antibodies and visualized using an enhanced chemiluminescence (ECL) procedure. The primary antibodies, such as those against LC3, p62, p-Akt (ser473), Akt, p-mTOR (ser2448), mTOR, p-p70S6K (Thr389), p70S6K, GRP78/Bip, p-eIF2α (ser51), eIF2α, p-JNK (thr183/tyr185), JNK, CHOP, Beclin 1, β-actin and GAPDH, that were employed in this study were purchased from Cell Signaling Technology (USA).

### Clonogenic survival assay

After irradiation, cell survival was analyzed using a colony formation assay as reported previously^38^. Cells were washed with PBS buffer, trypsinized and counted using a cell counter (Coulter), after which they were diluted with fresh medium and replated into Φ60 Petri dishes at approximately 100 surviving cells per dish. After 14 days of growth at 37 °C, the cells were fixed with methanol and acetic acid, stained with methylene blue, and the number of colonies with greater than 50 cells were counted as survivors. All of the experiments were repeated at least three times. The parameters of the survival curve, such as the α and β values, were obtained from the survival fraction (SF) data by curve fitting using the linear-quadratic (LQ) model^5^ as follows:


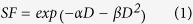


where *D* is the radiation dose delivered to the cells.

### Statistics

The data are represented as the mean ± standard deviation. Statistical analysis was conducted using an unpaired Student’s *t*-test. A difference was considered significant when *p* < 0.05.

## Additional Information

**How to cite this article**: Jin, X. *et al.* Carbon ions induce autophagy effectively through stimulating the unfolded protein response and subsequent inhibiting Akt phosphorylation in tumor cells. *Sci. Rep.* **5**, 13815; doi: 10.1038/srep13815 (2015).

## Supplementary Material

Supplementary Information
