# Supplementary material for: Carbon ions induce autophagy effectively through stimulating the unfolded protein response and subsequent inhibiting Akt phosphorylation in tumor cells
Source: Sci Rep. 2015 Sep 4;5:13815. doi: 10.1038/srep13815 (PMC4559768; doi:10.1038/srep13815)
Supplement: Supplementary Information [file srep13815-s1.pdf]

Supplementary Information

Carbon ions induce autophagy effectively through stimulating the unfolded protein response and subsequent inhibiting Akt phosphorylation in tumor cells

Xiaodong Jin [1,2], Feifei Li [1,2,3], Xiaogang Zheng [1,2,3], Yan Liu [1,2,3], Ryoichi Hirayama [4]

Xiongxiong Liu [1,2], Ping Li [1,2], Ting Zhao [1,2], Zhongying Dai [1,2], Qiang Li [1,2,*]

[1] Institute of Modern Physics, Chinese Academy of Sciences, Lanzhou 730000, China

[2] Key Laboratory of Heavy Ion Radiation Biology and Medicine of Chinese Academy of Sciences, Lanzhou 730000, China

[3] University of Chinese Academy of Sciences, Beijing 100049, China

[4] Research Center for Charged Particle Therapy, National Institute of Radiological Sciences, Anagawa 4-9-1, Inage-ku, Chiba 263-8555, Japan

*Corresponding author

Qiang Li, Phone: 86-931-4969316 Fax: +86-931-8272100 E-mail: liqiang@impcas.ac.cn

Supplementary Information: 2 pages, 1 table, 1 figure

Table S1 The parameters of survival curves of SHG44 cells treated differently

Treatment	α (Gy^{-1})	β (Gy^{-2})
X-rays	0.37	0.04
30 keV/μm carbon ions	0.61	-
75 keV/μm carbon ions	0.99	-
75 keV/μm carbon ions + CQ	1.24	-
75 keV/μm carbon ions + rapamycin	0.88	-

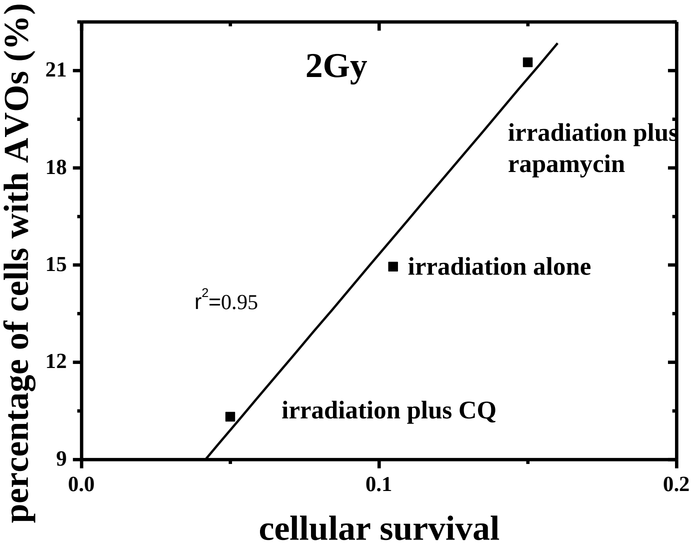

Figure S1 Percentage of SHG44 cells with AVOs 24 h after treatment with 2 Gy of radiation (75 keV/μm carbon ions), radiation plus CQ or radiation plus rapamycin as a function of the survival fraction. The data were fitted using the least-squares method.
